# P22 Using hospital electronic prescribing medicines administration (HEPMA) records to identify opportunities for improvement in IV to oral switch

**DOI:** 10.1093/jacamr/dlae217.026

**Published:** 2025-01-23

**Authors:** Jo McEwen, Kirsteen Hill, Heather Kennedy, Debbie Guthrie, Busi Mooka

**Affiliations:** NHS Tayside, Dundee, Scotland; NHS Tayside, Dundee, Scotland; NHS Tayside, Dundee, Scotland; NHS Tayside, Dundee, Scotland; NHS Tayside, Dundee, Scotland

## Abstract

**Background:**

The use of IV antibiotics in the inpatient setting has risen year on year since 2018. The higher use of IV antibiotics is detrimental to many elements within the health system such as increased requirement of nursing time, increased risk of skin and soft tissue infection, primary driver for *Staphylococcus aureus* bacteraemia (SAB), extended length of stay, increased cost and increased plastics waste. NHS Tayside is an outlier for both IV antibiotic use and SABs when compared with other Boards in Scotland, with up to 50% of local SABs resulting from peripheral vascular access devices. Historically, collecting Board-wide data was particularly challenging as manual data collection was resource and time intensive. Hospital Electronic Prescribing Medicines Administration (HEPMA) was introduced within NHS Tayside during mid 2023 initially focusing on acute inpatient areas. This allowed antimicrobial consumption data to be visible to the clinical teams.

**Objectives:**

With the recent introduction of HEPMA across the organization, the Antimicrobial Management Team used the live dashboard to determine whether opportunities for IV to Oral Switch (IVOST) existed.

**Methods:**

An IVOST point prevalence survey (PPS) was carried out across adult inpatient areas (except ICU) in two Acute hospitals. Patient level HEPMA records were screened to determine patients prescribed an antibiotic (IV or oral). Further investigation was carried out on IV prescriptions by reviewing Electronic Patient Records and blood results to determine suitability for IVOST by using the HOME (Haemodynamically stable, Oral route available, Markers of infection trending down, Excluding particular deep-seated infection) criteria. Appropriateness of prescription and compliance with policy were not measured during this PPS. Data were collected at ward level using Microsoft Forms to allow ward-, aggregated Clinical Care Group- and hospital-level feedback.

**Results:**

A total of 822 HEPMA records were screened. Of those records, 262 (32%) of patients were prescribed at least one IV or oral antibiotic. Where a patient was prescribed an antibiotic, it was identified that 50.4% (*n*=132) were prescribed these IV. Electronic records and blood results of patients prescribed an IV antibiotic were reviewed and cross matched with the HOME criteria where it was identified that 32% (*n*=42) of patients met the HOME criteria and could be considered for IVOST.

**Conclusions:**

The PPS was the first occasion where whole system antibiotic prescribing data could be examined to determine suitability for IVOST allowing for timely feedback to clinical teams, clinical care groups and strategic forums. The results enabled both widescale awareness campaigns and targeted promotional sessions on the benefits of IV to oral switch at patient, healthcare worker and organizational levels which were multi-professional in their delivery and attendance, acknowledging the changing landscape of prescribers within healthcare.

